# Partial repair versus superior capsular reconstruction: comparable mid-term outcomes in non-pseudoparalytic massive irreparable rotator cuff tears

**DOI:** 10.1186/s12891-026-10038-1

**Published:** 2026-06-20

**Authors:** Berhan Bayram, Muge Kirac, Tahir Koray Yozgatli, Alper Gamli, Arel Gereli, Baris Kocaoglu

**Affiliations:** 1 Department of Orthopedics and Traumatology, Acibadem Altunizade Hospital, Istanbul, Turkey; 2 Department of Orthopedics and Traumatology, Acibadem Mehmet Ali Aydinlar University School of Medicine, Icerenkoy Mah. Kayisdagi Cd. No: 32, Istanbul, 34638 Turkey; 3 Department of Orthopedics and Traumatology, Sarıyer Hamidiye Etfal Training and Research Hospital, Istanbul, Turkey

**Keywords:** Rotator cuff injury, Massive irreparable rotator cuff tear, Partial rotator cuff repair, Superior capsular reconstruction, Fascia latae autograft, Shoulder arthroscopy

## Abstract

**Hypothesis/background:**

Both partial rotator cuff repair (PRCR) and superior capsular reconstruction (SCR) are frequently performed arthroscopically to treat non-pseudoparalytic massive irreparable rotator cuff tears (MIRCT). The purpose of this study was to compare the clinical and radiological outcomes of PRCR and superior capsular reconstruction using tensor fascia latae autografts (SCRTF) in the treatment of non-pseudoparalytic MIRCT. The hypothesis was that both techniques would yield improved postoperative clinical and radiological outcomes with no significant difference between techniques.

**Methods:**

This retrospective comparative study included 40 patients (20 PRCR, 20 SCRTF) operated between 2017-2021. All patients were non-pseudoparalytic with at Goutallier grade 3 or greater fatty degeneration. Exclusion criteria were glenohumeral arthritis, cuff tear arthropathy, and pseudoparalysis. Clinical outcomes were assessed with VAS, ASES, and qDASH scores. Radiologic evaluation included measurement of the acromiohumeral distance (AHD). Pre- and postoperative values were compared within and between groups at a minimum of 32 months of follow-up.

**Results:**

Both groups demonstrated significant postoperative improvements in VAS, ASES, qDASH, and AHD compared with baseline (*p*<0.001). However, no statistically significant differences were observed between PRCR and SCRTF in clinical or radiological outcomes (*p*>0.05). Complications occurred in 3 patients (15%) in the SCRTF group (2 graft-site hematomas, one muscle herniation), whereas no complications were reported in the PRCR group; this difference was not statistically significant (*p*=0.23).

**Conclusion:**

Both PRCR and SCRTF provide satisfactory clinical and radiological outcomes in the treatment of non-pseudoparalytic MIRCT. While SCRTF carries a risk of graft-related complications, the overall effectiveness of both techniques appears comparable. For this reason, there might be no necessity to perform SCRTF in the treatment of non-pseudoparalytic MIRCT. Careful patient selection remains critical in optimizing outcomes.

**Level of evidence:**

Level III, retrospective comparative study.

## Introduction

Rotator cuff tears (RCTs) are common in adults, with a reported prevalence of 22.1% that increases with age [1]. RCTs can vary in severity, ranging from minor injuries to massive tears, which present a substantial challenge for orthopedic surgeons. Massive RCTs may lead to progressive atrophy and loss of rotator cuff muscles, often resulting in superior migration of the humeral head and degeneration of articular cartilage [2].

An irreparable tear refers to a massive RCT plus severe muscle atrophy and retraction. The incidence of massive irreparable rotator cuff tears (MIRCT) ranges from 12% to 30% of all RCTs. The decision-making in the management of MIRCT requires consideration of many variables, like age, activity level, tear size, fatty degeneration, affected tendons, retraction amount, presence of osteoarthritis, superior migration of the humeral head, and lastly, cuff tear arthropathy with pseudoparalysis [3]. These tears are complex injuries, and there are various approaches to their treatment. Whenever possible, preservation of the joint is an essential goal in the treatment of MIRCTs; the protection or restoration of the subscapularis and infraspinatus (anterior and posterior checkreins of the shoulder) is crucial. With a centered humeral head and preserved force couple, prevention of cuff tear arthropathy and pseudoparalysis can be possible. The two most commonly used procedures with this goal are partial rotator cuff repair (PRCR) and superior capsular reconstruction (SCR).

Burkhart first described PRCR in the early 1990s in the treatment of MIRCT. It appeared to be a favorable option, but there were concerns that it may not be successful in patients with severe muscle atrophy and fatty infiltration [4]. PRCR aims to restore the force couple between the subscapularis and infraspinatus tendons. The restoration of this force couple is hypothesized to provide shoulder function despite a defect in the supraspinatus tendon [4]. On the other hand, SCR was first described by Mihata in 2013 [5]. In this technique, the superior capsule is reconstructed with an autograft or an allograft, with its medial edge positioned on the glenoid and its lateral edge on the greater tuberosity [6]. This reconstruction aims to restore the superior stability of the glenohumeral joint, centralize the humeral head, and increase the function of the remaining rotator cuff muscles [7]. While both techniques aim to restore function to the shoulder joint, SCR carries the additional risk of graft-site morbidity when a tensor fascia latae autograft is used.

Both PRCR and SCR are frequently performed arthroscopically to treat non-pseudoparalytic MIRCT in patients without glenohumeral arthritis; however, few studies compare the two techniques and there is no consensus on the optimal mode of treatment. This study aimed to compare the clinical and radiological outcomes of PRCR and superior capsular reconstruction using tensor fascia latae autografts (SCRTF). The hypothesis was that both techniques would yield improved postoperative clinical and radiological outcomes with no significant difference between techniques.

## Materials and methods

### Patient selection and study design

This was a retrospective comparative study. The study was approved by the institutional review board (Acibadem University and Acibadem Healthcare Institutions Medical Research Ethics Committee (ATADEK) 2021-16/02). Patients who underwent arthroscopic PRCR or SCRTF for non-pseudoparalytic MIRCT at a single institution between January 2017 and May 2021 were included in the study. The study design, conduct, and reporting followed the STROBE statement for observational studies and adhered to the SAGER guidelines to ensure appropriate reporting of sex and gender considerations [8, 9].

Inclusion criteria were absence of pseudoparalysis in physical examination and MIRCT tears and > Grade 2 fatty degeneration of the supraspinatus tendon based on the Goutallier classification with or without subscapularis tears as determined by the preoperative magnetic resonance imaging [10]. The exclusion criteria were osteoarthritic changes in the shoulder joint, rheumatologic disease, neurologic disorder at the affected extremity, cuff tear arthropathy, pseudoparesis and pseudoparalysis. Patients were classified as having pseudoparesis if they demonstrated active forward elevation greater than 45° but less than 90°, and as having pseudoparalysis if active forward elevation was less than 45°, while maintaining full passive range of motion (ROM) [11].

### Surgical technique

All surgical procedures were performed at a single center by two fellowship-trained shoulder surgeons (A.G., B.K.). Standard posterior and anterior arthroscopic portals were used to assess intra-articular pathologies. In patients with preoperative findings of biceps-related shoulder pain and intraoperative findings of long head of the biceps tendon pathology, a tenotomy or tenodesis was performed, depending on the tendon’s condition, the patient’s age, and the surgeon’s preferences. In both groups, if a tear of the subscapularis tendon was present, intra-articular single row repair to the tendon footprint was performed by using a 5.5-mm SwiveLock knotless anchor (Arthrex, Naples, USA).

### PRCR technique

**Fig. 1:**
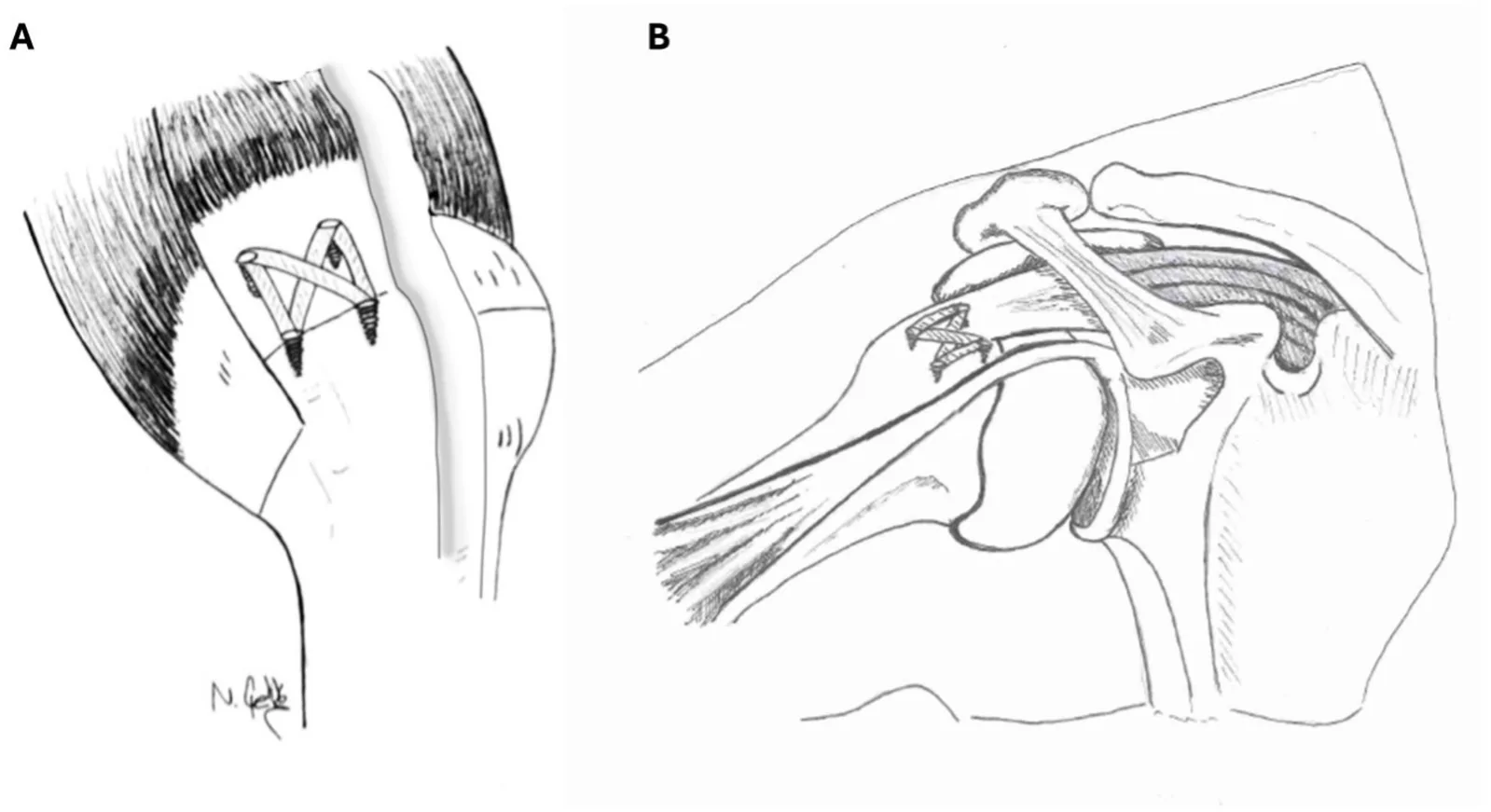
Illustration of the PRCR technique. PRCR: partial rotator cuff repair

Following the intra-articular assessment and procedures the subacromial space was accessed and additional portals were utilized as needed. The degenerated and retracted supraspinatus and infraspinatus tendons underwent debridement and mobilization from both the inferior-glenoid and superior-subacromial surfaces. Depending on the tear type, delamination, and retraction, margin convergence was performed, and non-viable tendon tissue was debrided with an arthroscopic shaver. The footprint was medialized and prepared by decortication with a burr device, and the posterosuperior cuff was then partially repaired from posterior to anterior direction with double or single row repair by 4.75 or 5.5-mm SwiveLock knotless anchors (Arthrex, Naples, USA). The PRCR technique is illustrated in Fig. 1.

### SCRTF technique

**Fig. 2:**
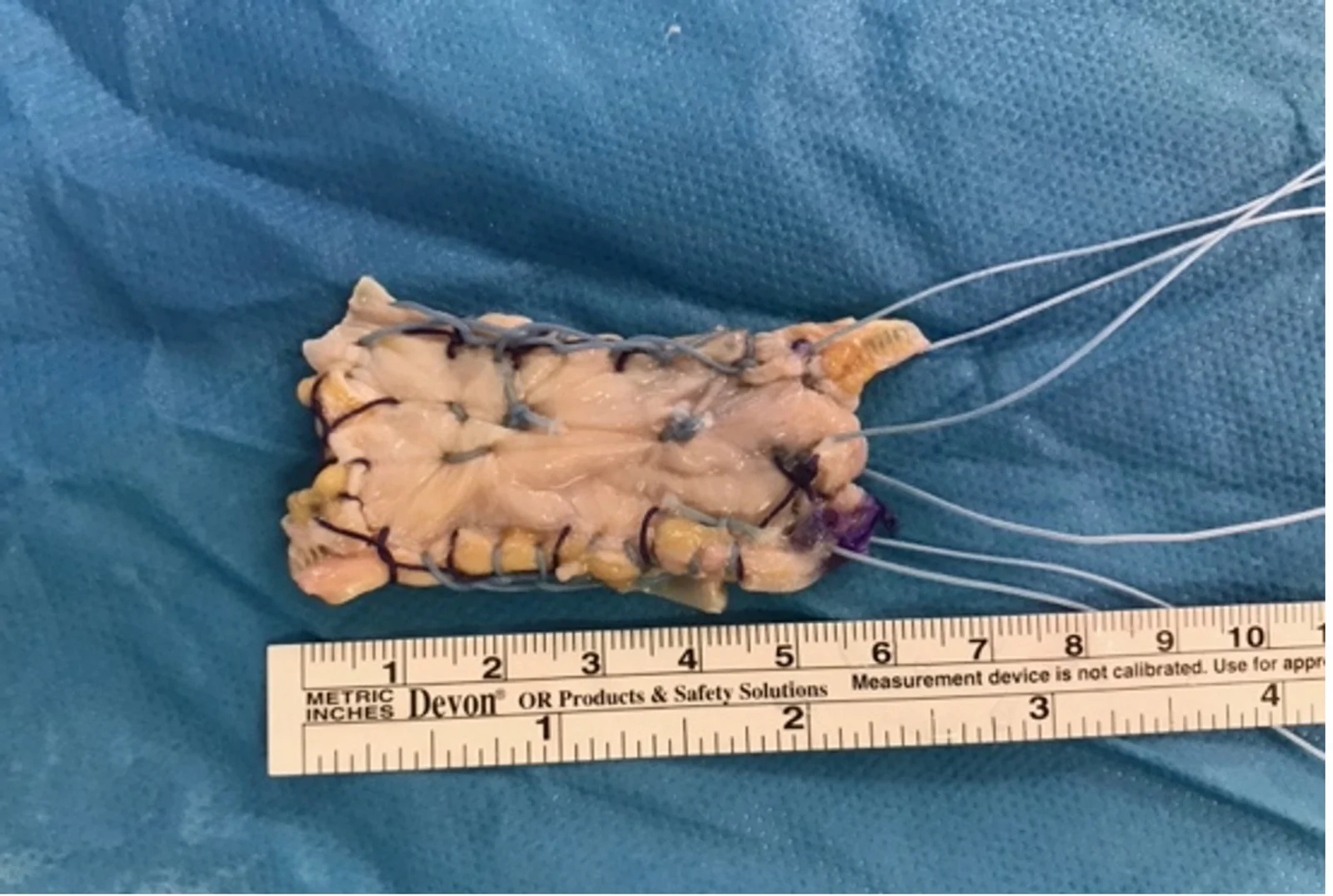
Preparation of the tensor fascia latae autograft for SCR. The graft was harvested from the lateral aspect of the thigh and folded three times to obtain adequate thickness. Final graft dimensions averaged 5.5 cm mediolaterally and 3.5 cm anteroposteriorly, with a minimum thickness of 8 mm. SCR: superior capsular reconstruction

In the SCRTF group, the tensor fascia latae graft was harvested from the lateral aspect of the thigh and was folded three times, resulting in an average graft size of 5.5 cm mediolaterally and 3.5 cm anteroposteriorly with a minimum graft thickness of 8 mm (Fig. 2). The meticulously prepared graft was inserted into the subacromial space through the lateral portal. The medial aspect of the fascia latae graft was fixed to the superior glenoid utilizing two knotless 3.5-mm PushLock anchors (Arthrex, Naples, USA). The fascia latae graft was subsequently fixed to the rotator cuff footprint utilizing a double-row technique characterized by a suture bridge configuration by using 4.75 or 5.5-mm SwiveLock knotless anchors (Arthrex, Naples, USA). Following graft fixation, side-to-side sutures were used to secure the infraspinatus tendon and the graft.

### Rehabilitation protocol

Both groups underwent the same postoperative rehabilitation protocol. An arm sling with an abduction pillow was used for 3 weeks postoperatively. Passive ROM exercises began in week 3, and active movements began in week 6. Strengthening exercises were initiated on week 12. Full return to activities was allowed after week 12 when patients achieved a full ROM.

### Patient-reported outcome measurements

Functional outcomes were assessed by administering the Visual Analog Scale (VAS) for pain, the American Shoulder and Elbow Surgeons (ASES) score, and the Quick Disabilities of the Arm, Shoulder, and Hand (qDASH) questionnaire, both prior to surgery and at the final follow-up visit. Literature-based minimal clinically important difference (MCID) values for ASES and qDASH (27.1 and 15, respectively) were used to evaluate changes in scores from the preoperative to the postoperative period [12, 13]. The acromiohumeral distance (AHD) was assessed both prior to and following the surgical intervention through true anteroposterior (AP) shoulder radiographs, which were acquired while the subject was positioned upright with standardized deltoid activation. The AP dimension and medial retraction of the compromised rotator cuff were quantitatively evaluated intraoperatively. All postoperative measurements were performed by an orthopaedic surgeon who was not part of the surgical team and was blinded to the patient’s surgical group.

### Statistical analysis

All statistical analyses were performed using NCSS (Number Cruncher Statistical System), 2007 (Kaysville, Utah, USA). Descriptive statistical methods (mean, standard deviation, median, frequency, ratio, minimum, and maximum) were used to evaluate the data. The Shapiro-Wilk test and graphical evaluations are used to determine whether the quantitative data were normally distributed. To compare the groups, Student’s t-test was used for normally distributed variables, whereas the Mann-Whitney U test was used for non-normally distributed variables. To compare intra-group pre- and post-operative outcomes, the Paired Samples t-test was used for normally distributed variables, whereas the Wilcoxon Signed Ranks test was used for non-normally distributed variables. Pearson’s chi-square test or Fisher’s exact test was used to compare qualitative data. A p-value less than 0.05 was evaluated as statistically significant.

A post hoc assessment of sample size adequacy was performed using literature-based minimal clinically important difference (MCID) values for the ASES score (27.1 points) and the qDASH score (15 points). Assuming a standard deviation of 10 points, a two-sided alpha level of 0.05, and a statistical power of 80%, the required sample size was estimated at 3 patients per group for the ASES score and 7 per group for the qDASH score. Given its smaller MCID and greater statistical sensitivity to detect clinically meaningful differences, the qDASH score was used as the reference outcome for sample size adequacy, and the larger calculated value was considered sufficient.

## Results

**Fig. 3:**
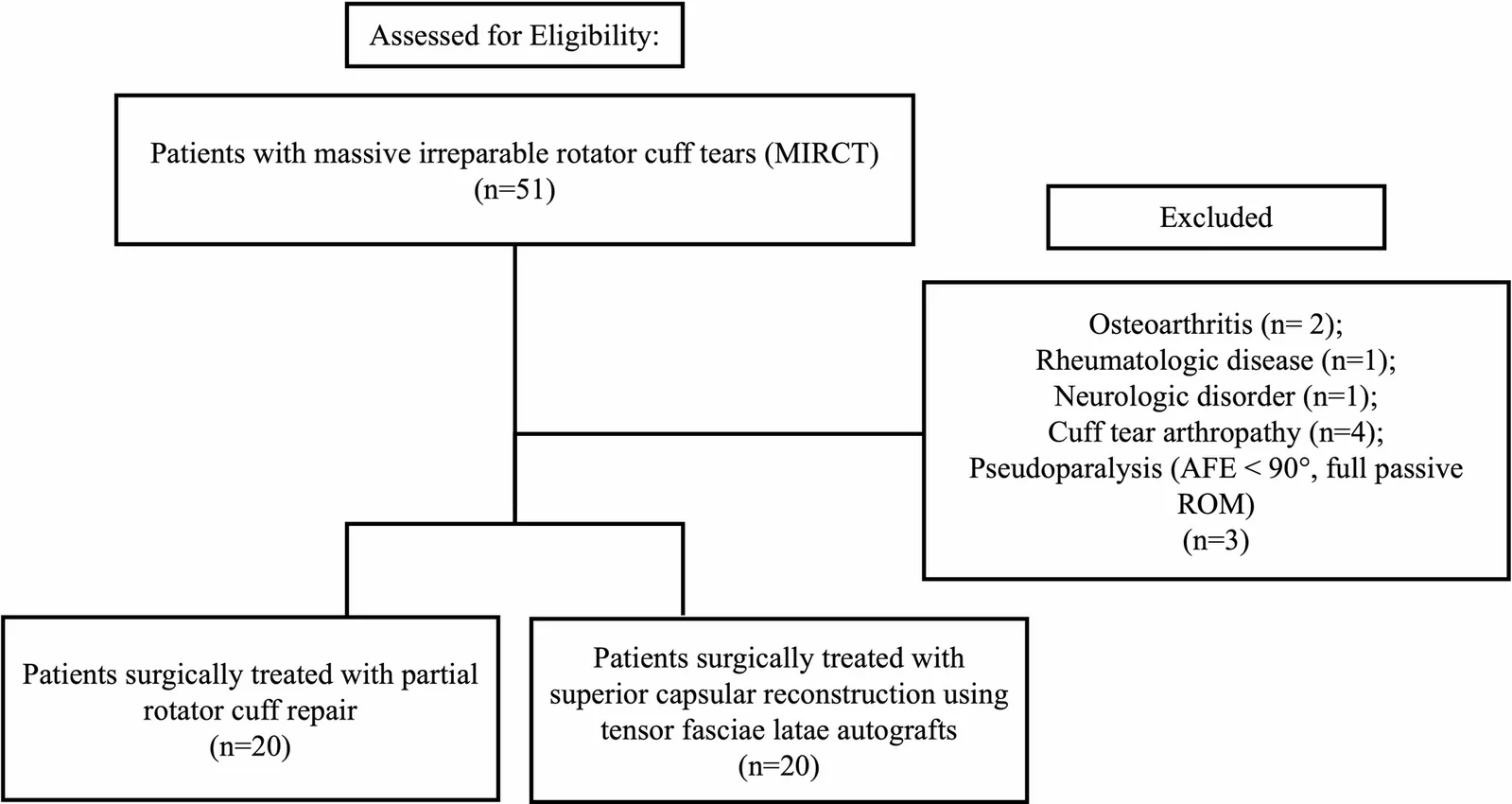
Flow diagram of patients included in the study

After applying the exclusion criteria, 40 patients were included in the study. There were 20 patients in the PRCR group, and 20 patients in the SCRTF group (Fig. 3). The groups were similar in terms of sex and age distributions (*p* > 0.05). Of the total cohort, 40% (*n* = 16) were female, and 60% (*n* = 24) were male. Eight (40%) of the patients in both groups were female, and 12 (60%) were male. The mean age of the patients was 60 ± 9 years. The mean age was 59 ± 12 years in the PRCR group and 61 ± 4 years in the SCRTF group (Table 1).

**Table 1:** Patient demographics

	PRCR (*n* = 20)	SCRTF (*n* = 20)	*p*- value
Male (*n*, %)	12 (60%)	12 (60%)	1
Female	8 (40%)	8 (40%)	1
Age (mean ± SD)	58.8 ± 11.6 (95% CI, 53.4–64.2)	61.3 ± 3.9 (95% CI, 59.5–63.1)	0.37
Follow up duration in months (mean ± SD)	52 ± 26 (95% CI, 39.2–63.9)	50 ± 9 (95% CI, 45.9–54.4)	Male (*n*, %)

No statistically significant differences were observed between the groups regarding age and gender distribution (p > 0.05)

*PRCR *Partial Rotator Cuff Repair, *SCRTF *Superior Capsular Reconstruction using Tensor Fascia Latae autografts, *n *number, *SD *standard deviation, *CI *confidence interval

The average follow-up duration was 52 ± 26 months in the PRCR cohort, and 50 ± 9 months in the SCRTF group. Pre- and postoperative AHD, forward flexion, external rotation at 90 degrees of abduction, VAS, ASES, and qDASH were similar (Table 2). Postoperatively, seventeen patients in both groups (PRCR: 17/20, 85% vs. SCR: 17/20, 85%, *p* = 1.00) achieved MCID in terms of improvements in ASES scores, while all patients achieved MCID in terms of qDASH scores (PRCR: 20/20, 100% vs. SCR: 20/20, 100%, *p* = 1.00).

**Table 2:** Pre-operative and post-operative AHD, forward flexion, external rotation at 90 degrees of abduction, VAS, ASES and qDASH values of PRCR and SCRTF groups

	Pre-Operative			Post-Operative		
	PRCR (*n* = 20)	SCRTF (*n* = 20)	*p*- value	PRCR (*n* = 20)	SCRTF (*n* = 20)	*p*- value
AHD (mm) (mean ± SD)	7.6 ± 0.8 (95% CI, 7.3–8.0)	7.8 ± 1.5 (95% CI, 7.1–8.5)	0.63	9.1 ± 0.8 (95% CI, 8.7–9.5)	9.2 ± 1.5 (95% CI, 8.4–9.9)	0.85
Forward Flexion (degrees) (mean ± SD)	140 ± 20 (95% CI, 131.7–147.3)	137 ± 16 (95% CI, 128.9–144.1)	0.57	165 ± 10 (95% CI, 159.9–169.1)	163 ± 10 (95% CI, 157.8–167.7)	0.55
External Rotation at 90 degrees of abduction (degrees) (mean ± SD)	37 ± 3 (95% CI, 35.9–38.7)	37 ± 3 (95% CI, 35.0–38.0)	1	57 ± 6 (95% CI, 53.8–59.2)	53 ± 8 (95% CI, 49.3–56.7)	0.08
External Rotation at Side (degrees) (mean ± SD)	52 ± 6 (95% CI, 49.4–55.1)	55 ± 8 (95% CI, 51.3–59.2)	0.19	63 ± 10 (95% CI, 58.0–67.0)	61 ± 9 (95% CI, 56.2–64.8)	0.51
VAS (mean ± SD)	8.5 ± 1.7 (95% CI, 7.7–9.3)	8.2 ± 1.9 (95% CI, 7.3–9.1)	0.60	0.8 ± 2.0 (95% CI, -0.1–1.7)	1.1 ± 2.4 (95% CI, 0.0–2.2)	0.67
ASES (mean ± SD)	42.5 ± 9.8 (95% CI, 37.9–47.1)	44.3 ± 10.8 (95% CI, 39.3–49.4)	0.58	90.8 ± 14.6 (95% CI, 84.0–97.6)	89.9 ± 14.1 (95% CI, 83.3–96.5)	0.84
qDASH (mean ± SD)	55.0 ± 3.7 (95% CI, 53.3–56.7)	53.9 ± 4.3 (95% CI, 51.9–55.9)	0.39	11.4 ± 18.4 (95% CI, 2.7–20.0)	11.7 ± 17.7 (95% CI, 3.4–20.0)	0.96

*PRCR *Partial Rotator Cuff Repair, *SCRTF *Superior Capsular Reconstruction using Tensor Fascia Latae autografts, *AHD *acromiohumeral distance, *VAS *Visual Analog Scale, *ASES *American Shoulder and Elbow Surgeons, *qDASH *quick Disabilities of the Arm, Shoulder, and Hand, *n *number, *mm *millimeters, *SD *standard deviation, *CI *confidence interval

In terms of postoperative complications, three patients (15%) in the SCRTF group developed complications (two graft-site hematomas, one muscle herniation). No complications were observed in the PRCR group. The difference was not statistically significant (*p* = 0.23).

## Discussion

The most important finding of this study is that both SCRTF and PRCR can provide satisfactory radiologic and patient-reported outcomes in the treatment of non-pseudoparalytic MIRCT, and there was no statistically significant difference in complication rates between the two groups. It is worth noting that SCRTF carries a risk of graft-related complications. Although the difference in complication rates between the groups was not statistically significant, the occurrence of complications in 15% of patients in SCRTF group compared with none in PRCR group may still be clinically relevant. Therefore, graft-site morbidity should be considered when selecting SCRTF for non-pseudoparetic and non-pseudoparalytic MIRCT patients.

MIRCTs are difficult injuries to treat, and various approaches are used. In an up-to-date review on the treatment of MIRCTs using a questionnaire developed by the European Society for Sports Traumatology, Knee Surgery and Arthroscopy (ESSKA) U45 committee with responses from 57 surgeons, it was emphasized that PRCR may be a good option in young patients [14] In a consensus formed by the ASES; it was emphasized that SCRTF is an unacceptable treatment in elderly patients with pseudoparalysis [15]. Conversely, in their systematic review, Dickerson et al. emphasized that SCRTF and reverse shoulder arthroplasty can both safely reverse pseudoparalysis that develops in MIRCT, but changes in ROM may affect this finding, so more studies with high scientific value are needed to make a clear conclusion [16]. Thus, there are several opinions in the scientific literature with some contradictions in terms of the treatment of MIRCT, and pseudoparalysis is a vital parameter to consider. The current study focused on MIRCT in non-psedoparetic and non-pseudoparalytic patients, defined as patients who can perform active forward elevation of 90 degrees or more in the shoulder joint.

Currently, SCRTF is primarily indicated for the treatment of non-pseudo-paralytic MIRCTs [5]. SCRTF aims to provide superior capsular coverage utilizing an autograft from the patient’s tensor fascia latae, which is harvested, folded, and prepared to ensure an adequately thick and strong graft. Ben et al. evaluated the efficacy of SCRTF in both failed rotator cuff repair and primary massive irreparable tears [17]. They stated that SCRTF improved clinical outcomes in both groups, but primary cases may yield better clinical outcomes than revision cases [17]. Tings et al. evaluated the factors affecting clinical outcomes in SCRTF in their study, including 13 studies, and found that better results can be obtained in young patients, patients with intact/repairable subscapularis, patients without acetabulization of the glenoid or glenohumeral arthritis, patients who play sports, and patients with an intact graft [18]. All these studies emphasize the importance of appropriate patient selection for SCRTF to be an effective treatment.

PRCR is a popular treatment option for MIRCT. It aims to repair the remaining rotator cuff in an attempt to provide improved shoulder function, even if the repair can’t be performed to cover the footprint of the torn tendon/tendons completely. In a systematic review by Hyde et al., who compared the efficacy of PRCR with arthroscopic debridement in MIRCT, both treatment methods yielded acceptable clinical outcomes, with no significant difference between them in short- and medium-term follow-up [19]. Malahias et al. evaluated whether PRCR is an effective treatment for MIRCT in a systematic review comprising 11 clinical studies and concluded that partial repair may be a safe and effective alternative in this patient group [20]. Chen et al. reported that arthroscopic PRCR with rotator interval shift in massive irreparable anterior L-shaped RCTs is an effective treatment method when short-term results are considered [21]. However, the authors noted that the preoperative acromiohumeral interval should not be less than 5 mm for PRCR patients, otherwise re-tears may occur [21]. Ishigaki et al. evaluated the 10 year follow-up results of patients who underwent PRCR for MIRCT stated that partial repair may be an effective treatment option [22] Similar to the current study, Schanda et al. retrospectively evaluated the results of patients who underwent arthroscopic SCRTF and arthroscopic partial repair or debridement [23]. In this study, 20 patients underwent SCRTF, 17 patients underwent PRCR, and 20 patients underwent arthroscopic debridement only. In all three treatment groups, there was improvement in PROs and ROM postoperatively, but there was no difference between the groups. However, it was reported that the SCRTF group had improved strength gain compared to the other groups, although the clinical significance of this gain is uncertain [23].

In the present study, SCRTF and PRCR treatments were compared with respect to clinical and radiological outcomes. Both groups improved radiologically and clinically after the operation, but there was no significant difference between the groups. No instances of progression to cuff tear arthropathy were detected in either cohort over a minimum of 32 weeks of follow-up. In all cases, the infraspinatus and subscapularis tendons were repaired as per the importance of the force couple of the shoulder joint, which Burkart et al. previously defined. Even in the absence of SCR or complete supraspinatus repair, a well-preserved subscapularis and infraspinatus tendon-muscle function could explain the improved outcomes in the current study. The findings from the current study align with the reports in the literature; it is also worth noting that the patient selection for both PRCR and SCRTF is critical. Previously, it was reported that outcomes of SCRTF were better for active patients with preserved joint integrity and intact or repairable subscapularis, and for PRCR, outcomes were better with a preserved acromiohumeral interval of at least 5 mm [18, 21]. In the current study, all patients had preserved active forward elevation, ensuring active glenohumeral mobility preoperatively, and infraspinatus and subscapularis functions were preserved or repaired, ensuring adequate force couple postoperatively. In this patient population, both surgical approaches provided adequate outcomes. There were three graft-related complications in the current study.

There were some limitations to the current study. First, the retrospective design of the study constitutes a limitation. Follow-up periods differed between groups, and long-term results could not be evaluated. Radiologic evaluations were performed with plain radiographs and may be less sensitive than advanced imaging methods. In addition, the fact that it was performed in a single center and by two surgeons limits the generalizability of the results. An additional limitation of the present study is that PRCR was compared with a single SCR technique, specifically SCR using a tensor fascia latae autograft. Accordingly, the findings should not be generalize to other SCR techniques, particularly SCR using the long head of the biceps tendon, which has recently gained popularity as an alternative reconstructive option.

## Conclusion

In the present study, all functional outcome scores demonstrated significant improvement in both the PRCR and SCRTF groups postoperatively. Among patients with MIRCT who maintained an intact force couple and exhibited neither pseudoparesis nor pseudoparalysis, no statistically significant differences were detected between PRCR and SCRTF in the evaluated functional and radiological outcomes. Given these findings, PRCR could be considered a reasonable surgical option for MIRCT patients over SCRTF due to the need for graft harvesting, the associated graft site-morbidity, and potential complications.

## Data Availability

The data that support the findings of this study are available from the corresponding author upon reasonable request.

## Abbreviations

AHD: Acromiohumeral distance
AP: Anteroposterior
ASES: American Shoulder and Elbow Surgeons
ESSKA: European Society for Sports Traumatology, Knee Surgery and Arthroscopy
MCID: Minimal clinically important difference
MIRCT: Massive irreparable rotator cuff tears
PRCR: Partial rotator cuff repair
qDASH: Quick Disabilities of the Arm, Shoulder, and Hand
RCTs: Rotator cuff tears
ROM: Range of motion
SCR: Superior capsular reconstruction
SCRTF: Superior capsular reconstruction using tensor fascia latae autograft
VAS: Visual Analog Scale
